# Neurovascular Alterations in Vascular Dementia: Emphasis on Risk Factors

**DOI:** 10.3389/fnagi.2021.727590

**Published:** 2021-09-10

**Authors:** Sarah Lecordier, Daniel Manrique-Castano, Yara El Moghrabi, Ayman ElAli

**Affiliations:** ^1^Neuroscience Axis, Research Center of CHU de Québec-Université Laval, Québec City, QC, Canada; ^2^Department of Psychiatry and Neuroscience, Faculty of Medicine, Université Laval, Québec City, QC, Canada

**Keywords:** vascular dementia (VaD), stroke, cerebral small vessel disease (cSVD), neurovascular abnormalities, blood-brain barrier, neuroinflammation, air pollution

## Abstract

Vascular dementia (VaD) constitutes the second most prevalent cause of dementia in the world after Alzheimer’s disease (AD). VaD regroups heterogeneous neurological conditions in which the decline of cognitive functions, including executive functions, is associated with structural and functional alterations in the cerebral vasculature. Among these cerebrovascular disorders, major stroke, and cerebral small vessel disease (cSVD) constitute the major risk factors for VaD. These conditions alter neurovascular functions leading to blood-brain barrier (BBB) deregulation, neurovascular coupling dysfunction, and inflammation. Accumulation of neurovascular impairments over time underlies the cognitive function decline associated with VaD. Furthermore, several vascular risk factors, such as hypertension, obesity, and diabetes have been shown to exacerbate neurovascular impairments and thus increase VaD prevalence. Importantly, air pollution constitutes an underestimated risk factor that triggers vascular dysfunction *via* inflammation and oxidative stress. The review summarizes the current knowledge related to the pathological mechanisms linking neurovascular impairments associated with stroke, cSVD, and vascular risk factors with a particular emphasis on air pollution, to VaD etiology and progression. Furthermore, the review discusses the major challenges to fully elucidate the pathobiology of VaD, as well as research directions to outline new therapeutic interventions.

## Introduction

Dementia affects nearly 50 million people worldwide, and the World Health Organization (WHO) estimates that this number will triple by 2050 (Patterson, 2018). Dementia is a heterogeneous neurodegenerative pathology that encompasses Alzheimer’s disease (AD), vascular dementia (VaD), Lewy body dementia (LBD), frontotemporal dementia (FTD), and Parkinson’s disease (PD). Although the overall prevalence of dementia is higher in aging men, its severity is more pronounced in aging females, a disparity that might implicate sex hormones (Appelros et al., 2009; Podcasy and Epperson, 2016; Poorthuis et al., 2017). VaD comes just after AD as a main cause of dementia, accounting for approximately 15–20% of dementia cases in the Western countries and could reach up to 30% in Asia and developing countries (Rizzi et al., 2014). Vascular deficiencies are now considered relevant contributors to mixed dementia (MxD), which accounts for 25–35% of all dementia cases (Jellinger, 2007; Rosa et al., 2020).

**Figure 1:**
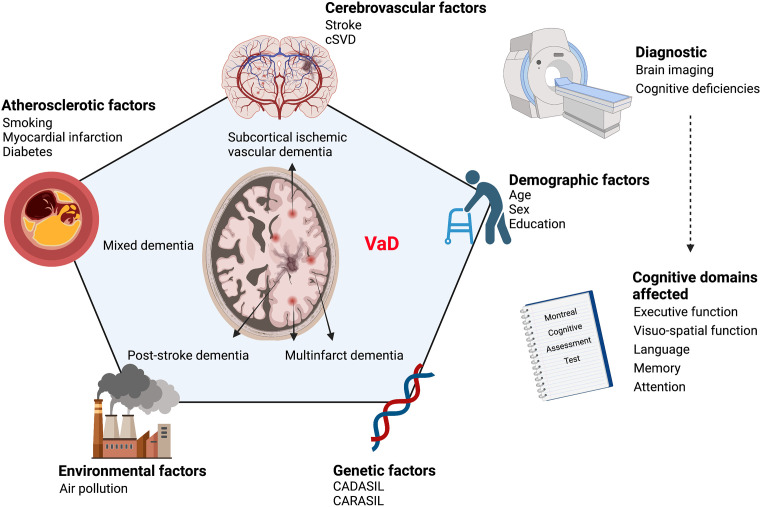
Scheme illustrating the continuum of risk factors that contribute to VaD etiology. VaD emerges as a conjunction of various risk factors affecting vascular homeostasis, namely cerebrovascular diseases, atherosclerosis, genetic and environmental factors. VaD diagnosis is based on the evaluation of cognitive deficiencies combined with neuroimaging to detect underlying vascular alterations. VaD, vascular dementia; CADASIL, cerebral autosomal dominant arteriopathy with subcortical infarcts and leukoencephalopathy; CARASIL, cerebral autosomal recessive arteriopathy with subcortical infarcts and leukoencephalopathy; cSVD, cerebral small vessel disease. Created with BioRender.com.

The Vascular Impairment of Cognition Classification Consensus Study (VICCCS) defines VaD as “clinically significant deficits in at least one cognitive domain comprising sensation, perception, motor skills and construction, attention and concentration, memory, executive functioning, processing speed and language/verbal speed, that are of sufficient severity to cause severe disruption of activities of daily living” (Sachdev et al., 2006; Andrianopoulos et al., 2017; Skrobot et al., 2018). The cognitive functions are assessed through the Montreal Cognitive Assessment Test which evaluates five cognitive domains; executive function, attention, memory, language, and visuospatial function (Pendlebury et al., 2012; Skrobot et al., 2018; Iadecola et al., 2019). Diagnosis of VaD is divided into two major research fields; cognitive tests and neuroimaging. Indeed, the diagnosis does not rely only on memory impairments, but it is supported by the presence of diverse cognitive deficits accompanied by diagnostic imaging evidencing cerebrovascular abnormalities such as brain atrophy, white matter hyperintensities, infarcts, and hemorrhages. Accordingly, four different subtypes arise: post-stroke dementia (PSD) in which dementia appears 6 months after stroke, subcortical ischemic vascular dementia (SIVaD), multi-infarct dementia, and MxD (Skrobot et al., 2018). The cognitive deficits associated with VaD are caused by structural and functional vascular abnormalities that are exacerbated with age. These abnormalities promote the emergence of chronic alterations in the neurovascular functions that underlie the etiology of cognitive decline observed in VaD (Figure 1).

VaD is tightly associated with several risk factors that can be categorized into four groups, which comprise: (i) cerebrovascular disease-related factors; (ii) atherosclerotic factors, such as smoking, myocardial infarction, diabetes mellitus, and hyperlipidemia; (iii) demographic factors, such as age, biological sex and education; and (iv) genetic factors, such as the emergence of mutations leading to vascular encephalopathies (Ritchie and Lovestone, 2002; Gorelick, 2004). Noteworthy, these vascular risk factors are now being recognized as clinical risk factors for AD pathology (O’Brien and Markus, 2014; Figure 1).

The cerebrovascular disease-related factors include cerebral tissue loss volume, bilateral cerebral infarction, strategic infarction, and white matter disease (WMD; Ritchie and Lovestone, 2002; Gorelick, 2004). Furthermore, hypertension was shown to be associated with larger white matter and smaller brain volumes, silent or strategical subcortical or cortical infarcts, and loss of volume in the thalamus or temporal lobe that are critical for cognitive functions (Ritchie and Lovestone, 2002; Gorelick, 2004). While age remains a principal risk factor for dementia, the presence of familial dementia history, and the epsilon 4 allele of apolipoprotein E (ApoE)4 susceptibility gene were recognized as an important risk factor for VaD (Ritchie and Lovestone, 2002; Gorelick, 2004). In addition, variables such as the female sex, various types of infection of lipid concentrations, history of head injury, head circumference, hormone replacement therapy (HRT), as well as thyroid dysfunction and preceding history of depression could interact with ApoE genotype and hence increase the risk of dementia (Ritchie and Lovestone, 2002; Gorelick, 2004). The genetic factors include vascular encephalopathies such as cerebral autosomal dominant arteriopathy with subcortical infarcts and leukoencephalopathy (CADASIL), autosomal recessive cerebral arteriopathy with subcortical infarcts and leukoencephalopathy (CARASIL), and potentially ApoE4 (Ritchie and Lovestone, 2002; Gorelick, 2004). Recently, environmental factors, namely air pollution, have been shown to constitute an important, yet underestimated, risk factor for dementia, inducing VaD and AD (Azarpazhooh and Hachinski, 2018; Béjot et al., 2018). Indeed, numerous studies have demonstrated that the elevated levels of air pollutants are directly linked to brain chronic inflammation and neurodegenerative diseases (Campbell et al., 2005; Schwartz et al., 2005; Calderón-Garcidueñas et al., 2007; Hartz et al., 2008; Block and Calderón-Garcidueñas, 2009; Mills et al., 2009; Rozemuller et al., 2012; Paul et al., 2019). Among air pollutants, ultrafine particles (UFPs) are particularly deleterious due to their ability to reach the brain where they act as inflammatory triggers and neurotoxins (Block and Calderón-Garcidueñas, 2009; Hameed et al., 2020).

As mentioned, VaD prevalence is strongly linked to cerebrovascular diseases, which essentially include stroke and cerebral small vessel disease (cSVD; Grinberg and Thal, 2010; Gorelick et al., 2011; Jellinger, 2013). Indeed, one patient in 10 has a stroke before developing a form of dementia that is not related to AD. In turn, cSVD was found in up to 62% of patients diagnosed with VaD, outlining a strong correlation between these pathologies (Gorelick et al., 2011; Venkat et al., 2015; van Veluw et al., 2017; Shih et al., 2018; Iadecola et al., 2019). Stroke and cSVD are characterized by the dysfunction of the neurovascular unit, which anatomically comprises sealed endothelial cells forming the blood-brain barrier (BBB), perivascular cells that include pericytes, and vascular smooth muscle cells (VSMCs), astrocytes, microglia, and neurons (Hermann and ElAli, 2012). The neurovascular unit integrates signals from the different neighboring cells to generate critical functions that include BBB maintenance, neurovascular coupling, vascular stability, and immunomodulation (Zlokovic, 2011; Hermann and ElAli, 2012). Neurovascular functions are impaired after stroke and cSVD leading to BBB dysfunction, neurovascular uncoupling, hypoperfusion, inflammation, and loss of neurons (Zlokovic, 2008, 2011; Guo and Lo, 2009; Moskowitz et al., 2010). These pathological events are at the origin of ischemic and hemorrhagic lesions, which strongly correlate with the cognitive deficits observed in VaD.

Despite being the second most common form of dementia after AD, little is known about the molecular and cellular mechanisms underlying the pathobiology of VaD. This gap in the literature is mainly due to disease heterogeneity in the clinical setup and the lack of an optimal experimental model that can accurately replicate most of the pathological events underlying the etiology and progression of the different forms of VaD. It is now established that accumulation of brain lesions over time mediated by neurovascular impairments constitutes a major contributor to the pathobiology of VaD in the elderly (Venkat et al., 2015; Corrada et al., 2016; Ince et al., 2017; Summers et al., 2017; van Veluw et al., 2017; Shih et al., 2018). The review summarizes the current knowledge related to the pathological mechanisms underlying the pathobiology of VaD with an emphasis on stroke, cSVD and risk factors with a focus on air pollution. We will discuss the challenges and research directions that might help in a better understanding of VaD pathobiology, thereby outlining new therapeutic interventions.

## Major Risk Factor-Mediated Mechanisms Implicated in VaD Pathobiology

### Stroke-Related Dementia

Ischemic or hemorrhagic strokes trigger major pathophysiological mechanisms that underlay VaD (Mijajlović et al., 2017). Indeed, epidemiological studies indicate that stroke history doubles the risk of dementia in the elderly (<65 years) and increases the incidence of early mortality (Savva and Stephan, 2010). Approximately 10% of patients exhibit signs of dementia before their first cerebrovascular accident, and another 10% manifest cognitive deficits soon after their first event (Desmond et al., 2002; Pendlebury and Rothwell, 2009). Particularly, recurrent stroke events raise the prevalence of dementia to 30%, constituting the most prominent causal factor of the disease (Pendlebury and Rothwell, 2009). Noteworthy, an examination of the association between stroke rates and dementia in the frame of the National Long-Term Care Survey (NLTCS) between 1984–2001 reported that the elevated incidence of post-stroke dementia is related to increased patient survival, due to clinical improvements in stroke management (Ukraintseva et al., 2006). Assessment of the neuropsychological parameters revealed that deterioration of the executive functions, abstraction, visual memory, and visuoconstruction constitute some of the most critical long-term cognitive disabilities observed in stroke patients (Sachdev et al., 2004). In contrast, praxis-gnosis, working memory, and language have been shown to be impacted to a lesser extent (Sachdev et al., 2004). The Sydney Stroke Study disclosed that in 50–85 years old patients diagnosed with VaD, the stroke volume and premorbid function were the most significant determinants of cognitive deterioration following the initial insult (Sachdev et al., 2006). It is remarkable to notice that in the last decade, stroke incidence increased by 23% in young adults aged between 35 and 50 years, especially because of the unhealthy lifestyle that meaningfully increased the rate of risk factors in this population, including smoking, hypertension, and obesity (Ekker et al., 2019; George, 2020). Due to the advances in acute stroke care, young stroke patients live longer and thus are at high risk of developing dementia at later stages (Pinter et al., 2019).

#### BBB Dysfunction

**Figure 2:**
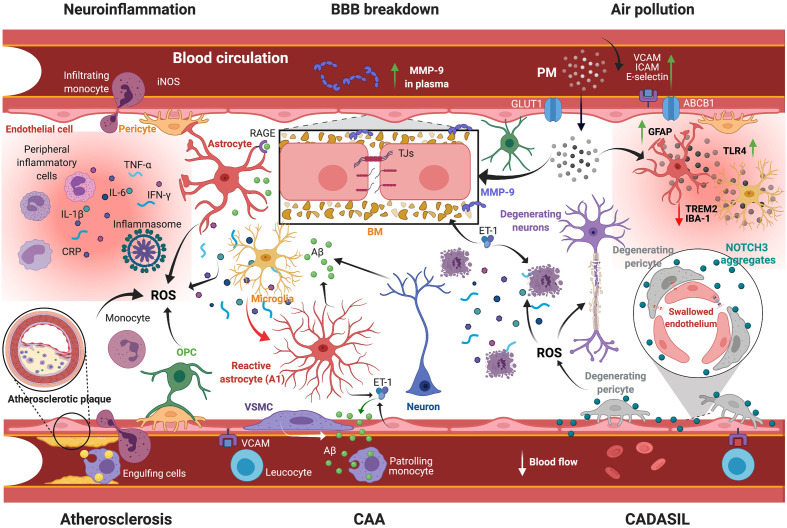
Scheme illustrating the mechanisms underlying VaD pathobiology. Several vascular risk factors are implicated in orchestrating pathological responses leading to VaD: (i) BBB breakdown involves the impairment of TJs and degradation of BM formed by ECM proteins *via* MMP-9 activity. Plasma MMP-9 levels constitute an effective prognostic marker for a poor neurological outcome; (ii) Post-stroke neuroinflammation comprises extravasation of peripheral immune cells and secretion of inflammatory mediators (e.g., CRP, TNF-α, IL-1β, IL-6, IFN-γ), as well as ROS generation and glial activation, accompanied by cerebral Aβ aggregation; (iii) Atherosclerosis comprises the accumulation of lipids and the calcification of immune cells into the intima, leading to vessel occlusion and hypoperfusion. This condition is associated with the generation of ROS that causes chronic inflammation; (iv) CAA is associated with the degeneration of VSMCs and vascular Aβ aggregation due to impaired clearance; (v) CADASIL is associated with NOTCH3 aggregation, causing endothelial cell swelling and pericyte degeneration and subsequently CBF impairment; and (vi) Exposure to air pollution, which implies PM infiltration into the brain, exacerbates BBB breakdown and neuroinflammation. VaD, vascular dementia; BBB, blood-brain barrier; TJs, tight junctions; BM, basement membrane; ECM, extracellular matrix proteins; MMP-9, matrix metalloproteinase-9; CRP, C-reactive protein; TNF-α, tumor necrosis factor-α; IL-1β/6, interleukin-1β/6; IFN-γ, interferon-γ; ROS, reactive oxygen species; Aβ, amyloid-β; CAA, cerebral amyloid angiopathy; VSMCs, vascular smooth muscle cell; CADASIL, cerebral autosomal dominant arteriopathy with subcortical infarcts and leukoencephalopathy; NOTCH3, neurogenic locus notch homolog protein-3; CBF, cerebral blood flow; PM, particulate matter; GLUT1, glucose transporter-1; ABCB1, ATP binding cassette subfamily B member-1; VCAM, vascular cell adhesion protein; ICAM, intercellular adhesion molecule; TLR4, Toll like receptor-4; TREM2, triggering receptor expressed on myeloid cells-2; GFAP, glial fibrillary acidic protein; RAGE, receptor for advanced glycation endproducts; ET-1, endothelin-1; iNOS, inducible nitric oxide synthase; OPC, oligodendrocyte progenitor cell. Created with BioRender.com.

Hallmark of stroke pathophysiology (Yang et al., 2019), BBB dysfunction constitutes a pivotal factor implicated in the initiation and exacerbation of the cascade of events leading to dementia (Zlokovic, 2011; Sachdev et al., 2014; Noe et al., 2020). Following primary injury, BBB breakdown allows the uncontrolled infiltration into the brain of blood-borne molecules, including plasma proteins, metabolites, neurotoxic compounds, and peripheral immune cells that contribute to secondary injury progression *via* edema formation, neuroinflammation, and glial reactivity (Halder and Milner, 2019; Koizumi et al., 2019) that aggravate the initial neurological deficits (Khanna et al., 2014; Jiang et al., 2018). The experimental findings indicate that acute BBB impairment is widely mediated by early inflammatory mediators, such as cytokines and chemokines, as well as oxidative stress, including reactive oxygen species (ROS) and reactive nitrosative species (RNS; Yang et al., 2019). The action of these substrates is further potentiated by matricellular proteins, proteoglycans, and metalloproteinases (MMPs) secreted in the extracellular space (Jones and Bouvier, 2014). Experimental and clinical studies revealed that MMP-9 significantly contributes to long-term BBB breakdown in several brain disorders, namely stroke and neurodegenerative diseases (Barr et al., 2010; Montagne et al., 2017; Underly et al., 2017; Figure 2).

Upon ischemic stroke, MMP-9 is secreted by the cells forming the neurovascular unit *via* regulation of the extracellular signal-regulated kinase-(ERK)-1/2) and the signal transducer and activator of transcription (STAT)-3 pathways, leading to the degradation of basal lamina/extracellular matrix (ECM) proteins, and the recruitment and extravasation of peripheral immune cells (Nishikawa et al., 2018; Jäkel et al., 2020). Interestingly, human brain studies showed that MMP-9 is implicated in the degradation of type IV collagen at the basal lamina, resulting in hemorrhagic transformations (Rosell et al., 2006, 2008), enhanced leukocyte infiltration, and poor neurological outcomes (Kim et al., 2016). In parallel, studies employing MMP-9^−/−^ mice showed that leukocyte-derived MMP-9 plays an essential role in mediating BBB dysfunction and is associated with elevated leukocyte transmigration that exacerbates the inflammatory signaling in the acute phase of stroke (Gidday et al., 2005). Furthermore, photothrombotic mouse models of cerebral ischemia have reported that BBB permeability at the level of the capillary is governed by pericytes exhibiting MMP-9 activation, which was later neutralized by specific MMP-9 inhibition (Underly et al., 2017). Elevated MMP-9 activity has been also reported to be associated with increased brain edema and IgG extravasation after ischemia in hyperlipidemic mice (ElAli et al., 2011). These observations indicate that hyperlipidemia exacerbates stroke-mediated BBB dysfunction, which could eventually aggravate dementia (Figure 2).

On an important note, stroke-induced MMP-9 expression has risen as an informative prognostic marker for a poor neurological outcome, increased mortality, and the emergence of typical signs of dementia (Zhong et al., 2017). Clinical investigations disclosed that high MMPs expression correlates with increased levels of albumin cerebrospinal fluid (CSF) in patients with vascular cognitive impairment (VCI) derived from SIVD, multiple strokes, and leukoaraiosis (Candelario-Jalil et al., 2011). In parallel, independently of the presence of vascular risk factors, elevated serum MMP-9 levels are associated with mild (25.6% of patients) and severe (27.4%) cognitive impairment 3 months following stroke according to the Mini-Mental State Examination and Montreal Cognitive Assessment (Zhong et al., 2018). Moreover, high MMP-9 levels in patients with cardioembolic stroke involving the middle cerebral artery (MCA) territory are related to large infarct volumes and poor behavioral scores based on the National Institutes of Health Stroke Scale (NIHSS; Montaner et al., 2001). Otherwise, the strong correlation between MMP-9 expression and the hyperintense acute reperfusion injury marker (HARM) has led to consider this protein as a revealing marker for BBB disruption (Barr et al., 2010). Furthermore, increased MMP-9 activity has been detected in the frontal and parietal cortex of postmortem human brains diagnosed with AD and patients exhibiting cognitive deficits (Bruno et al., 2009). MMP-9 enhanced expression is also notable in the CSF of AD patients, directly correlated with T-tau and P-tau levels (Stomrud et al., 2010). Importantly, a longitudinal study (4–10 years) enrolling AD and VaD patients revealed higher MMP-9 levels in the CSF of VaD patients compared to AD or controls (Adair et al., 2004). These findings imply that assessment of MMP-9 levels might constitute a potential strategy to distinguish between the different types of dementia.

Cell-based assays have demonstrated that exposure of pericytes to amyloid-β (Aβ)_42_ induced MMP-9 activity, which in turn ameliorated protein aggregation (Schultz et al., 2014). This outlines the presence of a direct pathological link between the molecular markers of dementia and MMP-9 activity, even though MMP-9 is an active substrate for Aβ degradation (Hernandez-Guillamon et al., 2015). Overall, current evidence suggests that MMP-9-mediated BBB dysfunction following stroke may constitute an early pathological mechanism that initiates the neurodegenerative cascades leading to cognitive deficits over time. Although MMP-9 inhibition has been proposed as a therapeutic strategy to attenuate BBB breakdown after cerebral ischemia (Dong et al., 2009; Chaturvedi and Kaczmarek, 2014), animal studies showed that MMP-9 is required for neurovascular remodeling and adaptation in the chronic phase after stroke (Zhao et al., 2006). Furthermore, MMP-9 is implicated in the clearance of various misfolded proteins involved in several neurodegenerative diseases, such as Aβ (Hernandez-Guillamon et al., 2015). The current knowledge implies that MMP-9 impact on BBB breakdown and neurodegeneration is time and context-dependent, which entails a contextualized modulation of protein expression/activity to preserve BBB integrity and attenuate cognitive deficits associated with stroke.

#### Post-stroke Neuroinflammation

Evidence obtained from AD studies outlined an important pathological link among chronic neuroinflammation, vascular damage, and cognitive decline in aged patients (Rhodin and Thomas, 2001; Kinney et al., 2018). Importantly, the neuroinflammatory responses associated with AD could be observed as well in other forms of dementia, including frontotemporal dementia (FTD; Bevan-Jones et al., 2020), PD (Caggiu et al., 2019), and VaD (Iadecola, 2013). Neuroinflammation plays a critical role in modulating tissue injury and repair after stroke. This process integrates various molecular and cellular mechanisms that comprise the release of inflammatory and oxidative stress mediators, glial reactivity, and peripheral immune cell activation and extravasation (Guruswamy and ElAli, 2017; Dzyubenko et al., 2018; Jayaraj et al., 2019). In this regard, uncontrolled microglial activation and the subsequent release of proinflammatory cytokines after stroke (Zhao et al., 2017) are strongly associated with demyelination and axonal loss (Sachdev et al., 2004).

It has been reported that in a rodent model of cerebral hypoperfusion, microglial activation *via* the complement (C)3-C3aR pathway, which is implicated in myelin phagocytosis, resulted in learning and memory deficits (Zhang et al., 2020). Interestingly, the cognitive impairments were attenuated by the genetic deletion of *c3ar1* or *via* the administration of SB290157, a potent C3aR antagonist (Zhang et al., 2020). Similarly, experimental investigations using cerebral ischemia have shown that fingolimod (FTY720), a potent agonist of sphingosine 1 phosphate (S1P), induced a microglial anti-inflammatory phenotype (M2 phenotype) through the activation of STAT-3 signaling pathway (Qin et al., 2017). Modulating microglial activation to adopt a protective M2 phenotype resulted in enhanced oligodendrocytogenesis and white matter integrity, and reduced cognitive deficiencies associated with working memory (Qin et al., 2017). Induction of severe chronic cerebral hypoperfusion (SCCH) in APP/PS1 mice that overproduced Aβ accelerated spatial learning and memory decline in 4-month adult animals. This was correlated to the accumulation of parenchymal Aβ plaques in the hippocampus and diminished activity of the ERK-1/2 pathway. APP/PS1 mice subjected to SCCH had higher levels of patrolling monocytes in peripheral blood. Interestingly, this model revealed that SCCH reduces microglial interaction with Aβ plaques in the hippocampus, denoting a reduced capacity for Aβ clearing in the brain parenchyma (Bordeleau et al., 2016). Alternatively, the release of prostaglandins, which act as inflammatory mediators upon stroke, has been shown to be associated with exacerbated Aβ-mediated cognitive decline and impaired synaptic plasticity (Kotilinek et al., 2008; Figure 2).

Loss of white matter integrity by hyper-reactive ramified and amoeboid microglia was also found in the posterior cingulate cortex of post-mortem brains of patients diagnosed with Down syndrome who are at higher risk of developing AD neuropathology (Martini et al., 2020). Furthermore, microglia in post-mortem AD brains exhibit accelerated aging and transcriptional alterations associated with the isoforms of ApoE, a protein broadly related to both dementia and cardiovascular disease (Srinivasan et al., 2020). In this regard, findings from subarachnoid hemorrhage in mice indicate that ApoE mediates protective effects following injury by inducing M1 microglial quiescence (Pang et al., 2018), suggesting that adequate lipid metabolism modulates neuroinflammation. Functional human brain investigations using positron emission tomography (PET) coupled to 11C-PK11195, which is an *in vivo* marker of activated microglia, have unraveled a progressive microglial activation and neuroinflammation, which were correlated with long-term (14 to 16 months) cognitive decline in AD patients (Malpetti et al., 2020). In this regard, activated microglia exhibiting a pro-inflammatory neurotoxic phenotype (M1 phenotype) trigger the activation of pro-inflammatory astrocytes (A1 astrocytes) *via* tumor necrosis factor (TNF)-α, interleukin (IL)-1α, and C1q cytokines (Liddelow et al., 2017). In turn, A1 reactive astrocytes exacerbate oligodendrocyte and neuronal death (Liddelow et al., 2017).

In line with these findings, an exaggerated astrocyte reactivity has been related to dementia and cognitive decline (Jo et al., 2014; Csipo et al., 2020). The evidence suggests that morbid neuroinflammatory responses maintained by A1 reactive astrocytes could result in mediating brain injury or age-related neurodegeneration and cognitive deficits. For instance, hippocampal astrocytes are susceptible to the upregulation of inflammatory-related genes and pathways, such as C3 and C4b, C-X-C motif chemokine ligand (CXCL)-10, and the peptidase inhibitor serine protease inhibitor A3N (SERPINA3N; Clarke et al., 2018). Interestingly, in a mouse model of familial Danish dementia (FDD), abundant A1 reactive astrocytes were detected in the brain, which correlated with the appearance of cerebral amyloid angiopathy (CAA), a disease characterized by the deposition of Aβ within the cerebral vasculature and a major risk of VaD. In this context, an increased number of astrocytes was observed in perivascular zones, accompanied by numerous cell branches and enhanced glial fibrillary acid protein (GFAP) expression (Taylor et al., 2020; Figure 2).

Several approaches have demonstrated that the usage of anti-inflammatory strategies could attenuate cognitive deficits associated with dementia. For instance, activation of the cannabinoid receptor 2 (CB_2_R) using different agonists promoted memory restitution through the reduction of oxidative stress and mitochondrial dysfunction (Jayant and Sharma, 2016). In line with these findings, cell-based assays have shown that CB_2_R activation stimulated the microglial release of IL-10, a key anti-inflammatory cytokine, *via* activation of ERK1/2, c-Jun N-terminal kinase (JNK), and mitogen-activated protein kinases (MAPKs) pathways, accompanied by the inhibition of the nuclear factor-κB (NF-κB) pathway (Correa et al., 2010). Likewise, administration of the CB_2_R agonist paeoniflorin (PF) ameliorated memory and learning deficits in mice, accompanied by induction of M2 cells and the release of anti-inflammatory mediators, such as transforming growth factor (TGF)-β1, and IL-10, instead of pro-inflammatory ones, such as TNF-α, IL-1β, and IL-6. This phenotypic switch is driven by the enhanced activity of phosphoinositide-3-kinase (PI3K/AKT) anti-inflammatory pathway and the inhibition of the mammalian target of rapamycin (mTOR)/NF-κB pro-inflammatory signaling (Luo et al., 2018). Experimental findings indicate that the inhibition of mTOR attenuated cognitive deficits, which were accompanied by a restoration of M1/M2 microglia phenotypic switch following cerebral hypoperfusion (Chen et al., 2016).

Remarkably, in a rodent model of VaD, it has been shown that acupuncture could attenuate inflammation by reducing TNF-α and Toll-like receptor (TLR)4 expression in microglia, and suppressing the myeloid differentiation factor (MyD88)/NF-κB pathway (Wang et al., 2020). Furthermore, pharmacological administration of PLX5622, a potent inhibitor of colony stimulating factor-1 receptor (CSF-1R) that is required for microglial cell survival, improved short-term memory in a rodent model of induced hypertension. This effect was accompanied by controlled microglia reactivity and preservation of BBB integrity (Kerkhofs et al., 2020). It has also been demonstrated that depletion of microglia *via* CSF1R inhibition prevented Aβ plaque development in the hippocampus of a mouse model of AD (Spangenberg et al., 2019). Attenuation of microglial reactivity *via* blockage of CCL-5 signaling also preserved BBB integrity in the context of systemic inflammation (Haruwaka et al., 2019). Taken together, there is strong evidence associating chronic uncontrolled neuroinflammatory responses after stroke to the emergence of long-term cognitive disabilities and dementia, mediated essentially by microglial reactivity. Therefore, strategies aiming to modulate microglial response by stimulating a protective phenotype might constitute a potential approach to attenuate VaD occurrence after stroke.

#### Stroke-Mediated Proteinopathies

Accumulation of Aβ in the brain constitutes a hallmark of AD pathogenesis (Chen et al., 2017). Early studies using human post-mortem brains revealed that amyloid precursor protein (APP) is not implicated exclusively in AD pathology, and its expression is as well induced in the brain after stroke (Cochran et al., 1991; Jendroska et al., 1997). For instance, mutant mice overexpressing APP exhibited a substantial reduction of cerebral blood flow (CBF) accompanied by larger infarcts after stroke, suggesting that APP exacerbated ischemic injury by impairing structural and functional vascular integrity (Zhang et al., 1997). Moreover, it has been shown that cerebral ischemia promotes APP deposition in the lesion core, the perilesional regions, as well as in the white matter areas exhibiting myelin loss (Nihashi et al., 2001; Zhan et al., 2015).

Endothelin (ET)-1 is a powerful vasoconstrictor synthesized by endothelial cells and reactive astrocytes, which have been shown to be implicated in ischemic stroke pathobiology as well as Aβ deposition. Examination of post-mortem human brains showed strong ET-1 expression in reactive astrocytes surrounding Aβ plaques (Hung et al., 2015). Furthermore, ET-1 overexpression in the acute phase after stroke has been involved in BBB disruption, glial reactivity, and neuronal death. Indeed, mutant mice exhibiting astrocytic ET-1 overexpression (GET-1 mice) experience severe memory and spatial learning deficits, associated with the upregulation of cleaved caspase-3, TNF-α, and IL-1β (Thiel et al., 2014; Hung et al., 2015). Using cell-based assays, ET-1 overexpression in reactive astrocytes has been shown to amplify Aβ production (Hung et al., 2015). Aβ accretion contributes to the development of cognitive deficits by impairing the receptor for advanced glycation endproducts (RAGE)-mediated Aβ clearance, which exacerbates inflammation, oxidative stress, and neurodegeneration (Min et al., 2020). These findings indicate that ET-1 upregulation after ischemic stroke is tightly associated with Aβ production and deposition and has considerable effects on excitotoxicity and BBB integrity. Furthermore, comorbid models of Aβ toxicity and cerebral ischemia have reported that Aβ deposition exacerbates ischemic damage. This condition leads to ventricular enlargement and striatal atrophy, morphological alterations in microglia, increased production of inflammatory mediators and enhanced glial communication *via* the gap junction proteins connexin (CX)-43. These observations are particularly important as ventricular enlargement was associated with deposition of neurofibrillary tangles and Aβ plaques, directly implicated in the pathogenesis of various forms of dementia (Amtul et al., 2015). Furthermore, evidence indicates that the interactions among lipoprotein-associated triggering receptor expressed on myeloid cells (TREM) 2 and apolipoproteins are involved in modulating microglia-mediated Aβ phagocytosis (Yeh et al., 2016), thus suggesting that Aβ clearance is associated with lipid metabolism (Figure 2). Likewise, it has been shown that exogenous administration of Aβ triggers tau phosphorylation and magnifies learning and memory deficits in animals subjected to cerebral ischemia (Song et al., 2013).

Finally, the generation of RNS, including peroxynitrite, revealed by the formation of 3-nitrotyrosine (3-NT), is increased in perivascular astrocytes and microglial cells after Aβ_42_ injection, strongly correlating with BBB leakage (Ryu and McLarnon, 2006). These findings suggest that Aβ pathology could trigger the release of reactive nitrogen species in astrocytes, directly undermining cerebrovascular integrity. Moreover, it has been demonstrated that Aβ binding to RAGE induces ROS production leading to loss of the tight junction proteins claudin-5, occludin, and zonula occludens (ZO)-1, as well as deficient endothelial cell function (Carrano et al., 2011). Overall, this bidirectional pathological crosstalk implies that exacerbated cognitive decline strongly emerges when cerebral injury and Aβ toxicity occur comorbidly.

### cSVD

cSVD comprises numerous pathologies impacting cerebral arteries, arterioles, venules, and capillaries, which are associated with diverse pathological and etiological processes (Østergaard et al., 2016; Staszewski et al., 2017; Li et al., 2018; Parkes et al., 2018). Six different types of cSVD are classified according to their etiology (Pantoni, 2010; Li et al., 2018). Atherosclerosis and sporadic and hereditary CAA are the most frequent forms. Recent reports outlined a significant increase in the number of genetic microangiopathies distinct from CAA such as CADASIL or Fabry’s disease (Razvi and Bone, 2006; Ballabio et al., 2007; Dichgans, 2007; Hara et al., 2009). Microangiopathies caused by inflammation or mediated by immunity are rare and characterized by the presence of inflammatory cells within the vasculature (Jennette and Falk, 1997), generally caused by mechanisms associated with systemic pathologies. Venous collagenosis is a pathologic thickening of the wall of veins and venules that are located near the lateral ventricles, thus leading to a smaller lumen and sometimes to an occlusion (Figure 2). Finally, post-radiation angiopathies are a side effect of cerebral irradiation that appears a few months to years after treatment. These angiopathies mainly affect small vessels of the white matter associated with fibrinoid necrosis, resulting in an increased thickness of the walls accompanied by a reduced diameter, which jointly could lead to a thrombotic occlusion (Dropcho, 1991).

#### cSVD Associated Parenchymal Pathology

cSVD refers to various and complex pathological and etiological processes. Therefore, the clinical manifestations depend on both the cause of the pathology and the affected brain territory. Among the most common symptoms are stroke-related manifestations, progressive cognitive deterioration, VaD, gait disturbance, sphincter dysfunction, and psychiatric disorders (van der Flier et al., 2005; Pantoni, 2010; Del Bene et al., 2013; Li et al., 2018; de Laat et al., 2011). cSVD is thought to constitute the major cause of vascular cognitive deficits and are responsible for up to 45% of dementia cases (Shi and Wardlaw, 2016; Li et al., 2018). Cognitive deficits are associated with impaired executive functions, decline in memory and attention, regression in verbal fluency, and delayed recall. These symptoms are accompanied by others that are not specific, including dizziness, trouble sleeping, tinnitus, and hearing loss. Moreover, neuropsychiatric symptoms can be observed, including hallucinations, agitation, depression, anxiety, disinhibition, apathy, irritability, and changes in appetite. Most of these manifestations are often accompanied by brain microbleeds. Cerebral microangiopathies are accountable for up to 20–30% of ischemic stroke as well as a considerable proportion of hemorrhage and encephalopathies caused by emboli, thrombosis, or stenosis of the vessel (Cai et al., 2015; Shi and Wardlaw, 2016; Regenhardt et al., 2019).

In addition to the cerebrovascular pathologies, cSVD exhibits a unique parenchymal pathology characterized by small subcortical infarcts, lacunar stroke, microbleeds, white matter hyperintensities (WMH), enlarged perivascular spaces, and brain atrophy detectable in imaging (van der Flier et al., 2005; de Laat et al., 2011; Del Bene et al., 2013; Wardlaw et al., 2013; Li et al., 2018; Regenhardt et al., 2018; Das et al., 2019). Small subcortical ischemic stroke is the result of severe tissue ischemia caused by the occlusion of a perforating arteriole. Patients either have typical stroke symptoms or a lesion visible only using neuroimaging approaches (Wardlaw et al., 2013; Li et al., 2018). Lesions can be anywhere in the brain and are round or ovoid and less than 20 mm in diameter (Smith et al., 2012; Brundel et al., 2012; van Veluw et al., 2017; Hartmann et al., 2018). They appear hyperintense in a diffusion-weighted image (DWI), hypointense on the map of apparent diffusion coefficients, and normal to hyperintense in fluid-attenuated inversion recovery (FLAIR)/T2 imaging (Okazaki et al., 2015; Potter et al., 2015; Li et al., 2018). DWI is the most sensitive technique currently used to detect ischemia a few hours after stroke onset. Recent infarcts will form a cavity characterized by morphological changes that include a reduction in volume and diameter within 90 days of the onset of the infarction (Moreau et al., 2012; Potter et al., 2015; Li et al., 2018). These infarcts can evolve in three different ways, namely lacuna, WMH without cavitation T2-weighted sequence, and finally, they could disappear without visible consequences in conventional magnetic resonance imaging (MRI). When a recent small subcortical ischemic stroke resolves into lacuna, it actually forms a fluid-filled cavity called a lacunar stroke and represents 40% of acute ischemic strokes. Lacunar insults are divided in two different categories; cavitated old infarcts and incomplete infarcts (Fisher, 1965; Lammie et al., 1998; Regenhardt et al., 2018). Old infarcts are pan-necrotic cavitation with scattered macrophages whereas incomplete infarcts are described as exhibiting loss of neurons and oligodendrocytes associated with invading CD68^+^ macrophages and reactive microglia in addition to reactive astrocytes that are found inside and around the lesion site (Merino and Hachinski, 2000; Brundel et al., 2012; Regenhardt et al., 2018).

The vascular damage can also develop into BBB leakage or cerebral microbleeds, which appear as small, round, and homogeneous hypointense foci on T2-weighted MRI and are mostly asymptomatic. They originate essentially from the rupture of a precapillary arteriole and are usually associated with vascular risk factor exposition or vascular Aβ deposition (Cordonnier et al., 2007; De Silva and Faraci, 2016; Shi and Wardlaw, 2016; Toth et al., 2017). The rupture is caused by various factors such as age, hypertension, cerebral ischemia, dementia, and cerebral amyloid angiopathy (CAA), and participates in cognitive deficits, dementia, and transient neurological deficits (Martinez-Ramirez et al., 2014; Shi and Wardlaw, 2016; Li et al., 2018). Microbleeds trigger proliferation and migration of microglia and astrocytes as well as monocyte recruitment (Liddelow et al., 2017). The immune cells release various inflammatory factors that impair neuronal function, as well as neurotransmitters that may be neurotoxic and interfere with neuronal circuitry to promote cognitive decline (Tancredi et al., 2000; Beattie et al., 2002; Rosidi et al., 2011; Donzis and Tronson, 2014).

The cerebral white matter is composed of myelinated axons, myelinating oligodendrocytes, oligodendrocyte precursor cells (OPCs), astrocytes, and microglia (Hase et al., 2018). WMH is common in older people and is a typical feature of cerebral microangiopathies, which are associated with BBB disruption, small white matter infarcts, glial activation, loss of oligodendrocytes, and demyelination caused by chronic diffuse hypoperfusion associated with a reduced CBF (Prins and Scheltens, 2015; Li et al., 2018). WMH is generally located within the white matter including the pons and brainstem but also in the deep gray matter. It is distributed symmetrically and bilaterally and appears hyperintense on FLAIR or T2 MRI. Importantly, WMH triples the risk of stroke, doubles the risk of dementia, and substantially increases the risk of death (Debette and Markus, 2010; Pantoni, 2010; Shi and Wardlaw, 2016). Symptoms develop insidiously and are associated essentially with cognitive impairments, dementia, and depression (Debette and Markus, 2010; Pantoni, 2010; Shi and Wardlaw, 2016).

The perivascular space is an extension of the subarachnoid space that surrounds the brain microvasculature. It is a liquid-filled space that cannot be detected by conventional imaging in a physiological context. When this space is widened, it often appears hyperintense on T2 MRI, hypointensity on T1 weighting, and sometimes hypointense on FLAIR (Aribisala et al., 2013; Shi and Wardlaw, 2016; Li et al., 2018). Finally, brain atrophy refers to a diminished brain volume on neuroimaging characterized by symmetrical or asymmetrical decreased total volume, increased ventricular volumes, enlarged superficial sulci, and decreased specific gray or white matter volumes (Mok et al., 2011). One main region affected is the hippocampus and is associated with cognitive decline (Muller et al., 2011; Jokinen et al., 2012).

#### Atherosclerosis

Atherosclerosis is an age-related condition that constitutes a major risk factor for cerebral microangiopathies. As its severity is increased by diabetes and hypertension, it is also called hypertensive microangiopathy (Tan et al., 2017; Li et al., 2018; Ter Telgte et al., 2018). The risk factors for atherosclerosis are also hyperlipidemia, smoking, and moderate to severe sleep apnea (Østergaard et al., 2016; Cannistraro et al., 2019). Atherosclerosis is characterized by a chronic inflammation associated with the deposition of low-density lipoproteins (LDL) within the vasculature, leading to its internalization by endothelial cells (Tabas et al., 2015), and resulting in the thickening and hardening of the arterial walls (Lusis, 2000; Shabir et al., 2018). Upon deposition, LDL undergoes oxidation by ROS to form oxidized (ox)-LDL, which further exacerbates the inflammatory response within the vasculature (Tabas et al., 2007). Indeed, by binding to vascular cell adhesion molecule (VCAM)-1 and P-selectin, monocytes can infiltrate the intima and differentiate into macrophages to engulf ox-LDL (Chistiakov et al., 2016). Macrophages, which are now called foam-cells due to the intracellular accumulation of lipids (Spann et al., 2012), accumulate and form stable fatty-streaks into the intima, and cells can calcify over time to slowly occlude the vessel (Alexander and Owens, 2012; Chistiakov et al., 2017). These pathological events occur in large to medium size arteries and lead to microbleeds, microinfarcts, as well as lipohyalinosis, characterized by the deposition of hyaline into the walls of connective tissue (Gorelick et al., 2011). This aspect is specific to the brain due to inflammation caused by ROS, ox-LDL, and gliosis involving astrocytes, OPCs, and microglia (Caplan, 2015). Lipohyalinosis fosters the infiltration of monocytes and T helper (TH)-1 lymphocytes that amplify the production of inflammatory mediators, such as TNF-α and interferon (INF)-γ (Stemme et al., 1995; Frostegård et al., 1999). Moreover, the assembly of inflammasomes can be promoted through the activation of nucleotide-binding oligomerization domain (NOD)-like receptor protein (NLRP)-3, stimulated by the formation of cholesterol crystals, caspase-1 and apoptosis-associated speck-like protein containing (ASC), caspase activation and recruitment domain (CARD; Weber and Noels, 2011). This results in IL-1β release, which in turn stimulates the release of IL-6 and C-reactive protein (CRP), implicated in the pathogenesis of atherosclerosis and thrombosis (Ridker et al., 2017; Figure 2).

Besides, the elevated levels of LDL combined with the low levels of high-density lipoprotein (HDL) play an important role in the pathogenesis of atherosclerosis and constitute as well a major risk factor for VaD (Hao and Friedman, 2014; Georgakis et al., 2020). Indeed, HDL exerts a protective role through its antioxidant properties and mediates beneficial effects on platelets and endothelial function, thus on coagulation and inflammation (Bandeali and Farmer, 2012). Furthermore, HDL interacts with triglyceride-rich lipoproteins, attenuating their deferential effects (Bandeali and Farmer, 2012). Moreover, it could contribute to the removal of cholesterol excess from the brain microvasculature through ApoE and heparin sulfate proteoglycans (Mulder and Terwel, 1998). HDL counteracts the inhibition of vessel relaxation caused by ox-LDL and decreases LDL peroxidation which affects cellular function and impairs membrane-bound receptors and enzymes (Braughler and Hall, 1992; Matsuda et al., 1993). Importantly, the impact of hyperlipidemia seems to be differentially modulated depending upon biological sex. Indeed, a recent study shows that females exhibit greater expression of genes related to neuroprotection in response to lipid stress compared to age-matching males (Nuthikattu et al., 2020). Finally, lipid derivatives are now being under the scope of researchers who are trying to unravel novel biomarkers to better understand and diagnose VaD pathology. Indeed, in an interesting recent study that aimed to discover lipid biomarkers in the context of VaD, it has been reported that patients with dementia exhibit low levels for ceramides, cholesterol esters, and phospholipids, and high levels of glycerides compared to controls (Liu et al., 2020). These observations indicate that lipid derivatives could indeed be used as novel diagnostic and prognostic biomarkers in VaD. However, more research is needed in this direction to validate the use of lipid derivatives as diagnostic and prognostic biomarkers in VaD.

Vascular damage caused by atherosclerosis can lead to microatheromas, microaneurysms, and even stenosis or obstruction of the vessel, impairing the mechanisms of blood flow autoregulation and leading to chronic cerebral hypoperfusion (Pantoni, 2010; Li et al., 2018). Importantly, occlusion of the cerebral arteries results in local ischemia or lacunar infarction (Kraft et al., 2017; Ter Telgte et al., 2018), while stenosis and hypoperfusion in the white matter cause incomplete ischemia lesions evidenced by neuroimaging as white matter hyperintensity (Rigsby et al., 2007). When the pathology affects the cerebral arterioles <50 μm in diameter, it is called small cerebrovascular atherosclerosis (Li et al., 2018).

#### Sporadic and Hereditary CAA

CAA is a chronic degenerative disease characterized by the loss of VSMCs and the accumulation into the vessel wall of eosinophilic hyaline material composed of soluble Aβ_40_ (Attems et al., 2011; Charidimou et al., 2017). CAA affects 50–60% of the elderly population affected by dementia, including 85–95% of AD cases (Sacco, 2000; Jellinger, 2002; Thal et al., 2003; Keage et al., 2009; Charidimou et al., 2017; Zhang et al., 2017; Li et al., 2018). The initial cognitive deficits associated with VaD could be explained by the particular sensitivity of the hippocampus and the cortex to CAA (Arvanitakis et al., 2011; Li et al., 2018). CAA is associated with changes in basal membrane (BM) composition and morphology that could predispose Aβ accumulation in the vessel even though the mechanism is not yet fully understood (Perlmutter et al., 1990, 1991; Su et al., 1992; Morris et al., 2014; Howe et al., 2020). Among the reported changes are BM thickening and degeneration, abnormal heparan sulfate proteoglycans (HSPGs) deposits, and irregular vasculature accompanied by increased collagen IV, fibronectin, agrin, and perlecan expression (Berzin et al., 2000; Farkas et al., 2000; Bourasset et al., 2009; Gama Sosa et al., 2010; Keable et al., 2016; Lepelletier et al., 2017; Magaki et al., 2018; Singh-Bains et al., 2019). Furthermore, vascular functional impairments are featured by BBB dysfunction caused by loss of endothelial cells, deregulation of mural cells mediated by oligomeric Aβ accumulation, as well as induction of astrocytosis with dystrophic endfeet surrounding BM Aβ deposits (Shimizu et al., 2009; de Jager et al., 2013; Giannoni et al., 2016; Yang et al., 2017; Magaki et al., 2018; Nortley et al., 2019). Two possible mechanisms for Aβ deposition have been proposed: (i) release of vascular Aβ from VSMCs directly into the vessel wall; and (ii) release of parenchymal Aβ by neurons which deposits afterward into the vessel wall (Davis et al., 2004; Herzig et al., 2004; Vidal et al., 2009; ElAli et al., 2013). In both cases, the protein accumulates due to a poor clearance towards the periphery (Davis et al., 2004; Herzig et al., 2004; Vidal et al., 2009; ElAli et al., 2013). Insufficient Aβ clearance can impair perivascular drainage pathways or diminish the ATP binding cassette subfamily B member-1 (ABCB1) and low-density lipoprotein receptor-related protein (LRP)1, a specialized endothelial-mediated active transport system implicated in Aβ mobilization from the brain into the blood circulation, namely (Deane et al., 2004; Herzig et al., 2006; Weller et al., 2008; Hawkes et al., 2011). Interestingly, LRP1 plays an important role in protecting against neurodegeneration. Indeed, LRP1 downregulation doesn’t only affect Aβ clearance but causes as well BBB breakdown through activation of MMP-9, thus leading to loss of neurons and cognitive deficits (Nikolakopoulou et al., 2021). Furthermore, recent evidence reveals that vascular Aβ could be engulfed and eliminated by circulating patrolling monocytes, which act as the housekeeper vascular homeostasis by surveying endothelial cells (Auffray et al., 2007; Carlin et al., 2013; Michaud et al., 2013; Thériault et al., 2015). In this regard, it has been demonstrated that patrolling monocytes located at the luminal wall internalize Aβ microaggregates that are diffusing from the parenchyma into the blood. Unfortunately, patrolling monocyte ability to phagocyte vascular Aβ in AD is defective, resulting in an overall increase of highly toxic Aβ_40_ and Aβ_42_ oligomers (Hallé et al., 2015; Gu et al., 2016). Moreover, chronic mild cerebral hypoperfusion impairs BBB functional properties and promotes the accumulation of circulating Aβ into the vessel wall, which initiates the cascade of parenchymal Aβ deposition (ElAli et al., 2013). Aβ accumulation and BM rearrangement trigger BBB breakdown, endorsing the formation of perivascular edema and the infiltration of toxic blood-derived substrates into the brain, which in turn contribute to the exacerbation of localized injuries and enlargement of the perivascular space (Holland et al., 2008; Hartz et al., 2012; Wardlaw et al., 2013; Li et al., 2018; Figure 2).

The overwhelming evidence is suggesting that the here mentioned vascular abnormalities leading to dementia reported in CAA occur as well in different forms of dementia, including AD and LBD (Salat et al., 2006; Okamoto et al., 2010; Soontornniyomkij et al., 2010; Arvanitakis et al., 2011; Love et al., 2014; Martinez-Ramirez et al., 2014; Reijmer et al., 2016; Li et al., 2018). This form of cSVD can be sporadic or of a genetic origin. For instance, a syndrome called hereditary brain hemorrhage with amyloidosis (HBHA) is associated with a mutation in the *APP* gene. This syndrome results in the deposition of misfolded amyloid fibrils in the walls of cerebral arterioles, which in turn activates a cascade of events leading to the development of CAA. The clinical phenotype develops between the ages of 45–65 years and is associated with intracerebral hemorrhages, WMH, multifocal lesions of a hemorrhagic and ischemic nature (Kamp et al., 2014; Marini et al., 2020). The presence of *ApoE4* allele, which constitutes the main risk factor for AD, has also been demonstrated to constitute an important risk factor for this form of cSVD (Hermann and ElAli, 2012). ApoE4 is a lipid-binding protein which plays an important role in lipoprotein metabolism as well as transport of triglycerides and cholesterol (Hirsch-Reinshagen et al., 2009). It binds to LDL, very-low-density lipoprotein (VLDL) debris, and some HDL *via* LRP (Bu, 2009; Leduc et al., 2010). ApoE4 can form complexes with Aβ and impairs Aβ through LRP thus attenuating its clearance and subsequently leading to its accumulation in the brain (Cho et al., 2001; Verghese et al., 2013). Moreover, ApoE4 increases the formation of Aβ oligomers, which are now well established to constitute the most neurotoxic form of Aβ (Hashimoto et al., 2012; Youmans et al., 2012). Interestingly, human pericytes of the prefrontal cortex and hippocampus of ApoE4 carriers exhibit increased activation of nuclear factor of activated T-cells (NFAT), which might account for CAA occurrence (Blanchard et al., 2020; Figure 2).

Moreover, non-APP sources of CAA exist and are caused by mutations of the BRI2 [i.e., integral membrane protein (ITM)2B] gene, essentially a codon stop mutation. Indeed, processing of the mutated form of BRI2 protein leads to the generation of 34-mer amyloid Bri (ABri) and amyloid Dan (ADan) peptides that accumulate in the brain, either to the ABri amyloid subunit or the AD amyloidogenic fragment. ABri and ADan are responsible for the Familial British (FBD) and Danish (FDD) dementia characterized among other pathological features by severe CAA (Vidal et al., 1999, 2000; Yamada and Naiki, 2012).

#### Genetic cSVD

Mutations in specific genes constitute the third most common cause of cSVD, among which the mutation of the *neurogenic locus notch homolog protein (NOTCH)3* gene is the better characterized (Cannistraro et al., 2019; Marini et al., 2020). NOTCH3 is a member of the transmembrane receptor NOTCH family, which is critically involved in developmental patterning, cell fate decisions, regulation of cell survival, and proliferation (Kopan and Ilagan, 2009; Bray, 2016; Baron, 2017; Hosseini-Alghaderi and Baron, 2020). During adulthood, NOTCH3 regulates stem cells and their lineages to promote tissue maintenance and repair. NOTCH3 is expressed by VSMCs and pericytes and plays a key role in regulating the crosstalk between the mural and endothelial cells. It controls the vascular tone and flow-mediated dilation *via* the modulation of the Ras homolog family member A (RHOA)/ Rho-associated protein kinase (ROCK) pathway in cerebral arteries (Joutel et al., 2000; Belin de Chantemèle et al., 2008; Li et al., 2009; Marini et al., 2020). However, the role of NOTCH3 is not restricted to the vasculature, since it is expressed in neural stem cells and is implicated in neuronal differentiation (Alunni et al., 2013; Kawai et al., 2017).

A mutation in the *NOTCH* *3 gene* is responsible for CADASIL, the most common autosomal dominant inherited cSVD (Louvi et al., 2006; Di Donato et al., 2017; Hosseini-Alghaderi and Baron, 2020; Marini et al., 2020). *NOTCH3* gene is affected essentially by missense mutations that lead to an odd number of cysteine residues located in the extracellular domain of the encoded receptor, and is associated with an early accumulation of the receptor’s extracellular domain containing aggregates in small vessels (Joutel et al., 2000, 2001; Monet-Leprêtre et al., 2013; Yamamoto et al., 2013). The function and activity of the NOTCH3 receptor are differently impacted by the mutations. However, the accumulation of extracellular domain containing aggregates in small vessels leads to mural cell degeneration *via* apoptosis or impaired proliferation (Joutel et al., 2000, 2001; Monet-Leprêtre et al., 2013; Yamamoto et al., 2013). Furthermore, the mutation itself causes profound morphological changes in pericytes, associated with dysfunctional mitochondria that could lead to oxidative and phosphorylation deficiencies, secondary lysosomes, and large cytoplasmic vesicles that result in cellular injury and autophagic apoptosis (de la Peña et al., 2001; Gu et al., 2012). This cascade of events cause neurovascular unit dysfunction characterized by detachment of astrocytic endfeet, destabilization of the vasculature, deregulation of vascular contractility, leakage of the BBB, and infiltration of toxic blood-born components into the brain parenchyma due to the decreased endothelial adherens junction protein, thus jointly resulting in diminished reactivity to CO_2_ (Ghosh et al., 2015; Figure 2).

Pericytes and endothelial cells are intimately interconnected through peg-and-socket junctions, which degenerate upon *NOTCH3* mutations. For instance, endothelial cells exhibit degenerative features, such as selective death or swelling, causing vessel stenosis or occlusion (Dziewulska and Lewandowska, 2012). Deregulation of pericyte-endothelial cells crosstalk causes cerebrovascular dysfunction that comprises reduced vascular density and impaired CBF (Tuominen et al., 2004; Lacombe et al., 2005; Miao et al., 2006; Gu et al., 2012; De Guio et al., 2014; Liu X.-Y. et al., 2015; Ihara and Yamamoto, 2016; Ping et al., 2019). Moreover, chronic cerebral hypoperfusion resulting from cerebrovascular dysfunction exacerbates pericyte degeneration, reduces pericyte coverage for the capillaries, and subsequently increases BBB permeability leading to white matter impairments and neuronal loss (Ueno et al., 2002; Bell et al., 2010; Montagne et al., 2018; Liu et al., 2019; Nikolakopoulou et al., 2019). BBB breakdown allows the infiltration into the parenchyma of toxic blood-born metabolites that accumulate around the vasculature, thus inducing macrophage, microglia, and T-cells activation and recruitment, which jointly promote axonal degeneration (Davalos et al., 2012; Ryu et al., 2015). Neuronal loss is mainly provoked by the secretion of pro-inflammatory mediators and the generation of ROS and RNS by pericytes within the perivascular space, which further exacerbates leukocyte adhesion and infiltration as well as microglial cell activation (Matsumoto et al., 2018; Erdener and Dalkara, 2019). Finally, the structural lesions within the white matter are worsened by the release of pericyte-derived bone morphogenetic protein (BMP)-4, which promotes astrogliosis (Uemura et al., 2018, 2020). Evidence of these pathological events could be detected using imaging approaches that indicate WMH, ischemic manifestations, subcortical hemorrhages, and microbleeds. A new sensitive assay was recently developed allowing pericyte injury detection in the CSF, a new technology that could serve as a diagnostic tool for WMD (Sweeney et al., 2020).

CADASIL develops gradually over time and the earliest symptoms appear on average around 30 years of age, usually 10 years earlier in women than men, and are manifested as migraines with aura (Guey et al., 2016; Di Donato et al., 2017). The migraine could also manifest with atypical attacks with basilar, hemiplegic, or prolonged aura and a few patients can even develop very severe attacks leading to confusion, fever, meningitis, or even coma that can mimic encephalopathy (Schon et al., 2003; Vahedi et al., 2004; Ragno et al., 2013; Tan and Markus, 2016; Drazyk et al., 2019). Adults between the age of 20 and 65 years are subject to transient ischemic attacks and stroke (Lesnik Oberstein et al., 2001). CADASIL is associated as well with some psychiatric manifestations, which include mood disturbances, severe depression, and schizophrenia (Lågas and Juvonen, 2001; Valenti et al., 2008, 2011; Noh et al., 2014; Ho and Mondry, 2015; Di Donato et al., 2017). Finally, 40% of symptomatic cases report apathy which drastically impacts the quality of life of CADASIL patients (Reyes et al., 2009). The final stage of CADASIL progression is dementia, but cognitive decline starts years before (Brookes et al., 2016).

A recessive form of CADASIL exists under the name of CARASIL. This form of hereditary cSVD is caused by the mutation of the *high-temperature requirement A serine peptidase 1* (*HTRA1*) gene which has two major functions, degrading various substrates and inhibiting TGF-β1 signaling pathway that is involved in various processes namely angiogenesis and BBB formation *via* pericyte-endothelial cell crosstalk (Oka et al., 2004; Hara et al., 2009; Shiga et al., 2011; Akhtar-Schaefer et al., 2019; Kandasamy et al., 2020). HTRA1 is expressed in various brain cells comprising endothelial cells and VSMCs (De Luca et al., 2003; Oka et al., 2004; Campioni et al., 2010; Tennstaedt et al., 2012; Tiaden and Richards, 2013). Loss of HTRA1 function results in increased TGF-β1 availability and thereby signaling, leading to vascular fibrosis and extracellular matrix synthesis, which jointly cause microvascular degeneration, CBF reduction, and neurogenesis alterations (Wyss-Coray et al., 2000; Tarkowski et al., 2002; Gaertner et al., 2005; Yamamoto et al., 2011; Zhang et al., 2012; Martinez-Canabal et al., 2013; Beaufort et al., 2014; Friedrich et al., 2015). Moreover, the mutation has been shown to be associated with impaired pericyte proliferation, accumulation of protein within the vessel walls, MMPs activity, and BBB permeability (Joutel et al., 2016; Baron-Menguy et al., 2017; Ikawati et al., 2018; Marini et al., 2020). Cognitive decline begins early compared with CADASIL, as well as gait disturbances, lower back, pain and alopecia (Shiga et al., 2011; Marini et al., 2020).

Fabry’s disease is an X-inherited rare disorder that belongs to the family of lysosomal storage diseases and is caused by a mutation in the *α-galactosidase (GAL)A* gene that encodes for α-GAL enzyme that plays a key role in sphingolipid metabolism (El-Abassi et al., 2014). The mutation causes deficiencies in α-GAL activity that results in the accumulation of sphingolipids in various organs and tissues including the vessels (Rolfs et al., 2013). The cerebrovascular complications in Fabry’s disease arise from peripheral neuropathy and are associated with mild to severe headache, vertigo, transient ischemic attacks, ischemic stroke, intracerebral hemorrhage, and VaD (Okeda and Nisihara, 2008). Furthermore, deposition of toxic metabolites within the vasculature and VSMCs lead to ischemia, vessel stenosis, occlusion, and dilation with local changes in CBF (Shimotori et al., 2008). The disease is more severe in men compared to women and is often present with infantile neuropathy, gastrointestinal symptoms, corneal opacity, hearing loss, and angiokeratoma (Sims et al., 2009; Schiffmann, 2015; Marini et al., 2020). Brain structural damage and symptoms exacerbate with age.

Collagen IV, which exists as a heterodimer derived from the transcription of *COL4A1* and *COL4A2* genes, is an essential component of the vascular BM. Mutations in these two genes are associated with microangiopathies in several organs (Germain et al., 2019; Marini et al., 2020). More precisely, *COL4A1* mutation is responsible for ocular, renal, muscular, and cerebral deficits (Vahedi and Alamowitch, 2011; Marini et al., 2020). Furthermore, cerebral microangiopathies have been shown to affect half of the carriers of this mutation. WMH, dilation of the perivascular spaces, lacunar infarctions, and microbleeds have been reported as well (Vahedi and Alamowitch, 2011). Pontine autosomal dominant microangiopathy and leukoencephalopathy (PADMAL) syndrome is a specific form of cerebral microangiopathies associated with the *COL4A1* mutation (Verdura et al., 2016), and is associated with an overexpression of the gene with the absence of protein misfolding. Patients with this syndrome have dysarthria, ataxia, and stroke as well as mood disorders and dementia. On the other hand, *COL4A2* mutation is associated with an increased prevalence of lacunar ischemic stroke and deep intracerebral hemorrhages (Verdura et al., 2016). In contrast to *COL4A1* mutation, *COL4A2* mutation impairs the trimerization of collagen IV due to defects in the α-helix structure, which cause BM instability, loss of vascular wall integrity, and increased BBB permeability, mediated by the intra- and extracellular accumulation of deficient collagen IV (Kuo et al., 2012; Meuwissen et al., 2015; Verdura et al., 2016; Zhang et al., 2017; Malik et al., 2018; Germain et al., 2019).

Forkhead Box C1 (FOXC1) is highly expressed in pericytes. Its expression plays an important role in controlling endothelial cell proliferation and vascular stability. *FOXC1* mutation reproduces some of the events reported upon *COL4A1* mutation, especially the ischemic infarctions, and cerebral microangiopathies that lead to WMH, which are visible in neuroimaging (French et al., 2014). The retinal vasculopathy with cerebral leukodystrophy (RVCL) syndrome includes three pathological conditions: (i) cerebral retinal vasculopathy (CRV); (ii) hereditary vascular retinopathy (HRV); and (iii) hereditary endotheliopathy with retinopathy, nephropathy, and stroke (HERNS). Patients with these syndromes possess a mutation in three prime repair exonuclease (TREX)-1 that encodes for a DNA exonuclease (Stam et al., 2016). This mutation causes a defect in apoptosis and INF signaling (Rice et al., 2015; Marini et al., 2020). All characteristics of the cerebral microangiopathies are found in RVCL patients who report the following symptoms: neurological deficits, migraines, cognitive deficits, psychiatric disorders, and seizures (Stam et al., 2016; Marini et al., 2020).

## Environmental Risk Factors: Air Pollution

The increasing interaction with the environmental risk factors associated with human activities has a significant impact on health due to the exposure to various hazardous pollutants. Currently, environmental factors are directly implicated in the etiology and progression of diverse pathologies, including brain diseases. Indoor and outdoor air pollution is among the environmental factors that play a particularly important role in the deterioration of vascular health (Block and Calderón-Garcidueñas, 2009). Indeed, air pollution, which is defined as the release of an amalgam of pollutants into the atmosphere, has been reported to increase the prevalence of cardiovascular, cerebrovascular, and respiratory diseases, as well as cancer (Campbell et al., 2005; Schwartz et al., 2005; Calderón-Garcidueñas et al., 2007; Hartz et al., 2008; Block and Calderón-Garcidueñas, 2009; Mills et al., 2009; Rozemuller et al., 2012; Cho et al., 2018; Paul et al., 2019). Epidemiological studies have indicated that nearly one-third of the global stroke burden and about one-fifth of the global dementia burden, including VaD, are attributable to air pollution (Feigin et al., 2016; Azarpazhooh and Hachinski, 2018; Béjot et al., 2018). Furthermore, numerous studies have outlined a link between high levels of air pollutants, chronic brain inflammation, and neurodegeneration (Campbell et al., 2005; Schwartz et al., 2005; Calderón-Garcidueñas et al., 2007; Hartz et al., 2008; Block and Calderón-Garcidueñas, 2009; Mills et al., 2009; Rozemuller et al., 2012; Paul et al., 2019). These effects are mainly attributable to the exposure to fine particulate matter (PM), and more precisely to PM of 2.5 microns or less in diameter (PM_2.5_). Indeed, the experimental findings have indicated that UFPs could reach the brain through different routes, including the intranasal cavity, where they act as an inflammatory mediators, thus deregulating the function of cells forming the neurovascular unit (Oberdörster et al., 2004; Peters et al., 2006). In particular, exposure to PM leads to impaired olfactory function, one of the initial atypical symptoms that emerge in individuals affected by different forms of dementia (Campbell et al., 2005; Schwartz et al., 2005; Calderón-Garcidueñas et al., 2007; Hartz et al., 2008; Block and Calderón-Garcidueñas, 2009; Mills et al., 2009; Rozemuller et al., 2012; Paul et al., 2019). The correlation between air pollution and dementia, including VaD and AD, was highlighted in various epidemiological studies relying mostly on cohort studies in polluted regions (Åström et al., 2021). The Betula cohort revealed an association of dementia incidence, AD in particular, with traffic-related air pollution (TRAP; Oudin et al., 2019). Moreover, studies have found that exposure of the elderly to air pollution, notably PM_10_ and PM_2.5_ was associated with cognitive decline (Wu et al., 2015). In an interesting case-control study, it was reported that elevated long-term PM_10_ levels were associated with a significantly increased risk of AD and VaD prevalence in the elderly. A dose-response relationship between PM_10_ exposure and the risk of AD and VaD was reported (Wu et al., 2015).

### Impact on Endothelial Functions

Various studies have investigated the impact of air pollutants, and more specifically PM, on endothelial functions. For instance, exposure of isolated rat brain capillaries to diesel exhaust particles (DEP) altered BBB function through oxidative stress generation and proinflammatory cytokine production, which jointly induced the expression of adhesion molecules that exacerbate infiltration of immune cells into the brain (Hartz et al., 2008). Exposure to DEP deregulated the expression of several transporters and receptors that are critically involved in BBB functionality namely ABCB1, multidrug-resistance associated proteins (MRP)1, MRP2, MRP4, breast cancer resistance protein (BCRP), glucose transporter (GLUT)1, and the metabolizing enzyme glutathione S-transferase (GST)π (Hartz et al., 2008). Several reports have demonstrated an increased BBB permeability following exposure to a mixed vehicular emission, translated essentially by deregulation of the TJs (Rojas et al., 2011; Oppenheim et al., 2013; Bernardi et al., 2021). For instance, human brain microvascular endothelial cells in culture exposed to nanoparticles of aluminum oxide exhibit reduced cell viability, altered mitochondrial function, increased oxidative stress, and diminished expression of the TJs proteins claudin-5 and occludin (Chen et al., 2008; Block and Calderón-Garcidueñas, 2009). Interestingly, epidemiological studies have outlined a strong correlation between air pollution and neuroinflammation in highly exposed residents (Calderón-Garcidueñas et al., 2008). Indeed, these studies reported an upregulation of some inflammatory markers, such as expression of cyclooxygenase (COX)-2 and IL-1β, as well as infiltration of immune cells into the olfactory bulb (OB), frontal cortex, substantia nigrae, and vagus nerves (Calderón-Garcidueñas et al., 2008). Importantly, most of the inflammatory responses were concentrated at the vasculature translated by activation of NF-κB pathway in brain endothelial cells, accompanied by oxidative stress, Aβ_42_ immunoreactivity, trafficking of inflammatory cells into the perivascular space, and an altered BBB (Calderón-Garcidueñas et al., 2008). In line with these observations, direct exposure of brain endothelial cells in culture to PM_2.5_ deregulated TJs and increased permeability and monocyte transmigration across the endothelial monolayer (Liu F. et al., 2015). In addition, exposure to both PM_2.5_ and PM_10_ induced the activation of endothelial cells accompanied by an enhanced adhesion of U937 monocytic cells to the endothelial monolayer (Montiel-Dávalos et al., 2007). Interestingly, exposure to PM_2.5_ also induced ICAM-1 expression, whereas exposure to PM_10_ induced expression of E-selectin and P-selectin (Montiel-Dávalos et al., 2007; Figure 2).

*In vivo* experiments in which mice were exposed to a mixed vehicular emission, a combination of gasoline and diesel engine exhausts, the animals exhibited altered BBB integrity through the deregulation of the TJs protein, namely claudin-5 and occludin (Oppenheim et al., 2013). This was accompanied by an augmentation of inducible nitric oxide synthase (iNOS) levels, an increase in the production of IL-1β in the parenchyma, and deregulation of ABCB1 transport activity (Oppenheim et al., 2013). Moreover, exposure to PM *in vitro* and *in vivo* has been shown to stimulate the re-localization of the TJs protein ZO-1 from the cell membrane and reduce its protein level (Wang et al., 2012). Importantly, PM mediated the intracellular mobilization of calcium (Ca^2+^) dependently upon ROS, activating calpain that is implicated in ZO-1 degradation and disruption of the endothelial barrier (Wang et al., 2012). Using a 3D human *in vitro* BBB model, indoor nanoscale particulate matter (INPM) was shown to translocate across the BBB and accentuate inflammation by inducing ROS (Li et al., 2020). This induction was followed by abnormal nuclear reactor factor (NRF)-2 expression and a disruption of the kelch ECH associating protein (KEAP)-1/antioxidant response elements (ARE) pathway which is involved in supporting cells to overcome stress (Li et al., 2020). Interestingly, exposure of rodents to urban PM increased the levels of ET-1 mRNA and reduced TNF-α mRNA levels in the cerebral hemisphere and the pituitary gland. These results suggest that the cerebrovascular effects of urban pollutants are associated with the modulation of gene expression involved in the regulation of vasoconstriction in the brain and pituitary gland (Benatti et al., 1993; Thomson et al., 2007).

Exposure to PM_2.5_ has been shown to increase the prevalence of carotid artery stenosis (CAS), a well-established risk factor for ischemic stroke, correlating with an increased BBB permeability (Newman et al., 2015; Szarmach et al., 2017). In line with these observations, a strong association between air pollution with systemic brain inflammation was revealed in children living in polluted areas, associated with short-term memory deficits, prefrontal WMH, and BBB disruption (Calderón-Garcidueñas et al., 2016). The same study reported a leaking vascular network, degeneration of pericytes, VSMCs, and endothelial cells, thickening of the BM, and reduced perivascular astroglial coverage in the prefrontal white matter of dog brains (Calderón-Garcidueñas et al., 2016). Exposure to PM_2.5_ has been demonstrated as well to accelerate atherosclerosis development through induction of vascular dysfunction as well as promotion of coagulopathies, which were accompanied by a strong inflammatory response and lipid abnormalities (Liang et al., 2020). Finally, exposure of ApoE-deficient mice to TRAP, mixed vehicle emissions, induced the cerebral expression of ICAM-1 and the release of pro-inflammatory mediators, such as TNF-α and IL-1β (Adivi et al., 2021).

### Impact on the Dynamics of Astrocytes and Oligodendrocytes

Evidence indicates that astrocytes respond to PM in a context-dependent manner (Allen et al., 2014). For instance, early postnatal exposure to ambient UFPs decreased GFAP immunoreactivity in male subjects and increased GFAP expression as well as other neuroinflammation markers in females (Allen et al., 2014). Exposure of male and female rodents during gestation and early postnatal development to TRAP attenuated astrogliosis specifically in the dentate gyrus (DG) associated with reduced GFAP immunoreactivity, which remained unchanged in CA1 and CA3 regions (Patten et al., 2020). Previous findings have shown that maternal exposure to carbon black nanoparticles (CB-NP) induced astrogliosis in the cortex of rodents, affecting the interaction of the astrocyte endfeet with the endothelium and perivascular macrophages (Onoda et al., 2017). Interestingly, intranasal delivery of PM_2.5_ to male rodents subjected to ischemic stroke exacerbated astrocytic reactivity *via* GFAP activation and iNOS induction, aggravating post-stroke neurobehavioral impairments (Chen et al., 2016). In line with these results, rodents exposed to PM for a prolonged period exhibited altered neuronal and astrocytic functions *via* impairment of mitochondrial activity (Araújo et al., 2019). Likewise, exposure of rodents to natural air pollution sources, such as volcanic-derived particles, increased GFAP immunoreactivity in astrocytes (Camarinho et al., 2019; Navarro et al., 2021). Moreover, exposure of rodents to low doses of CB-NP induced endoplasmic reticulum (ER) stress in perivascular macrophages and reactive astrocytes, specifically around the vasculature of offspring animals, associated with the accumulation of β-sheet rich misfolded proteins (Onoda et al., 2020). Cell-based assays have shown that exposure of astrocytes to PM activated janus kinase (JAK)-2/STAT-3 and p38/JNK/ERK pathways in reactive astrocytes triggering iNOS induction and IL-1β production (Li et al., 2016). In this regard, PM has been reported to increase the expression and release of proinflammatory mediators through activation of the NF-κB signaling pathway (Li et al., 1999; Gómez-Budia et al., 2020).

Furthermore, exposure to UFPs altered adult OPCs turnover and survival of mature oligodendrocytes (OLs), accompanied by increased oxidative stress and decreased total antioxidant capacities (TAC) that impair the remyelination capacity of the brain (Kim J. Y. et al., 2020). Furthermore, prenatal exposure to concentrated ambient particles (CAPs), which were defined as UFPs, promoted a premature maturational shift in OLs in the corpus callosum (CC), followed by hypermyelination (Klocke et al., 2018). Interestingly, females showed significant alterations in oligodendrocytogenesis in the CC (Klocke et al., 2018). Finally, in a mouse model of lysolecithin-induced demyelination of the subcortical white matter, exposure to PM_2.5_ hampered remyelination and disrupted oligodendroglia differentiation (Parolisi et al., 2021).

### Impact on Microglial Reactivity and Consequences on Neurons

In response to air pollution, microglia are activated through the upregulation of several proinflammatory mediators that affect neuronal function (Gómez-Budia et al., 2020). Microglia respond to air pollution by adopting an amoeboid shape *in vitro* (Cole et al., 2016; Roqué et al., 2016; Mumaw et al., 2017), as well as *in vivo* (Araújo et al., 2019). Indeed, mice exposed to DEP exhibited reduced adult neurogenesis through activation of microglia (Coburn et al., 2018). In contrast, attenuating microglial reactivity hampered neuroinflammation and oxidative stress (Coburn et al., 2018). Cell-based assays showed that exposure of BV-2 immortalized microglial cell to PM_2.5_ increased mRNA expression of various proinflammatory markers, namely IL-6, IL-1β, TNF-α, iNOS, COX-2, TREM2, and TLR2/4, while reducing mRNA expression of key anti-inflammatory markers, such as IL-10 and Arginase (ARG)-1 (Kim R.-E. et al., 2020). In parallel, exposure of rodents to DEP for a long period triggered the activation of microglia in the nuclei of the solitary tract (NTS), accompanied by ER dilation and mitochondrial vacuolization in the medulla, hence outlining structural alterations of the neuronal network (Chen et al., 2021). Interestingly, rodents exposed to DEP for a long period showed an induced ionized calcium-binding adaptor molecule (IBA)-1 expression in the brain (Levesque et al., 2011). Interestingly, microglial reactivity was accompanied by an increased mRNA expression of TNF-α, IL-6, and macrophage inflammatory protein (MCP)-1α in the midbrain, cortex, and OB, while IL-1β was expressed particularly in the midbrain (Levesque et al., 2011). Mice exposed to PM also triggered the induction of mRNA expression of TLR4, MyD88, TNF-α, and tumor necrosis factor receptor (TNFR)-2 in microglia (Woodward et al., 2017a). Activation of the latter was further confirmed through an increased intracellular expression of inflammatory mediators, such as COX-2, NF-κB, prostaglandin E2 (PGE2), and iNOS both *in vitro* and *in vivo* (Babadjouni et al., 2018; Chen et al., 2018). Using cell-based assays, TNF-α induction in microglia upon PM exposure inhibited neurite outgrowth (Cheng et al., 2016). Inhibition of NRF-2 activity prior to exposure of BV-2 microglial cells to PM_2.5_ attenuated cell viability, induced ROS generation, and stimulated NF-κB pathway, outlining NRF-2 role in mitigating PM deleterious effects (Chen et al., 2018).

Importantly, *in vivo* experiments showed that exposure to DEP in TREM2^−/−^ mice accentuated IL-1β expression (Greve et al., 2020). PM has been demonstrated to impact neuron-glial crosstalk. Acute exposure of adult mice to PM_2.5_ increased the levels of lipoperoxidation and proinflammatory cytokines in the brain and activated microglia, accompanied by reduced neurogenesis in the subgranular zone (SGZ) and subventricular zone (SVZ; Bernardi et al., 2021). Neuronal cell cultures exhibited reduced viability upon exposure to PM_2.5_ associated with increased release of glutamate (Liu F. et al., 2015). Prior treatment of cells with the *N*-methyl-D-aspartate (NMDA) receptor mitigated PM_2.5_-mediated neuronal loss (Liu F. et al., 2015). Moreover, neuronal cultures displayed dopaminergic neurotoxicity upon exposure to DEP only in presence of microglia, which was associated with elevated levels of ROS (Block et al., 2004). Co-culture of neurons and microglia exposed to PM_2.5_ in presence of oligomeric oAβ exacerbated IL-1β and ROS release aggravating oAβ-induced neuronal injury and inflammation (Wang et al., 2018). PM have been shown to directly impact neuronal function. For instance, exposure of rodents to nano PM caused hippocampal neurite atrophy and decreased expression of myelin basic protein (MBP), accompanied by increased TNF-α mRNA expression (Woodward et al., 2017b). In this regard, epidemiological studies have outlined a strong correlation between the levels of PM in air and neuronal chromatolysis and satellitosis in exposed dogs, associated with cortical neurons degeneration, and neurofibrillary tangle formation (Calderón-Garcidueñas et al., 2002).

Interestingly, epidemiological studies comprising elderly women revealed that residence in places contaminated with high levels of fine PM increases the risks for global cognitive decline and all-cause of dementia by 81 and 92%, respectively, with stronger adverse effects in ApoE4 carriers (Cacciottolo et al., 2017). Experimental findings obtained from female AD mouse models (5xFAD; Familial AD) expressing either ApoE3 or ApoE4 mice that were exposed to urban nano PM for 15 weeks showed increased Aβ plaques and soluble Aβ oligomers, which were associated with neuronal changes in the hippocampus (Cacciottolo et al., 2017). These findings were confirmed *in vitro* upon exposure of neuroblastoma cells (N2a-APP/swe) to nano PM translated by an enhanced pro-amyloidogenic processing of the APP, explaining the elevated cerebral Aβ production (Cacciottolo et al., 2017). Furthermore, long-term exposure to ambient air pollution was found to be associated with rapid cognitive decline in aged adults, where ApoE4 carriers exhibited the fastest cognitive decline (Kulick et al., 2020; Figure 2).

Further *in vivo* experimental investigations implicating exposure to TRAP nano PM showed an increased production of Aβ peptides, associated with oxidative damage (Cacciottolo et al., 2020). Indeed, exposure of J20-APPswe mice, an AD mouse model, to nano PM for 150 h revealed exacerbated lipid oxidation and pro-amyloidogenic processing of APP in lipid raft fractions compared to controls (Cacciottolo et al., 2020). These observations were further confirmed *in vitro* using N2a-APPswe cells exposed to nano PM (Cacciottolo et al., 2020). Importantly, the link between air pollution and HDL was highlighted in the Multi-Ethnic Study of Atherosclerosis Air Pollution (MESA Air) study showing that exposure to air pollution was significantly associated with low levels of HDL (Bell et al., 2017). In this regard, ApoE-deficient mice exposed for 2 weeks to DEP, exhibited high systemic pro-oxidant effects associated with dysfunctional HDL (Yin et al., 2013). These observations would indicate that PM exposure could be responsible for the attenuated HDL protective effects against atherosclerosis (Yin et al., 2013).

## Conclusion and Perspectives

Through this review, we aimed to highlight the structural and functional neurovascular alterations underlying VaD pathobiology. The epidemiological, clinical, and experimental investigations are indicating that the long-term outcomes of early pathophysiological events impacting neurovascular functions upon cerebrovascular disorders have major consequences on imitating a cascade of events that lead to VaD. Moreover, the recent findings are outlining air pollution as a major vascular risk factor that is directly implicated in promoting neurovascular impairments associated with VaD. Given the heterogeneity of cerebrovascular disorders, which include stroke, genetic and sporadic microangiopathies, combined with the diverse effects of environmental factors, a better understating of the short- and long-term remodeling processes at the neurovascular unit is urgently required to allow getting new insights into VaD pathobiology. Such knowledge will allow the identification of key “targetable” mechanisms for therapeutic purposes.

Recent findings are suggesting that BBB disruption occurs in some cases for a prolonged period beyond the acute and subacute phases after stroke (Bernardo-Castro et al., 2020). A prolonged, yet subtle, disruption of the BBB could trigger a cascade of events that lead to VaD. For instance, high expression levels of MMPs, the main mediator of BBB disruption, are significantly associated with cognitive deficits (Yang and Rosenberg, 2011). However, targeting MMPs is a double-edged sword as while preserving the BBB in the acute and sub-acute phase, its inhibition impairs neurovascular adaptation in the chronic phase, thus impeding brain plasticity (Yang and Rosenberg, 2011). More promising appears to be the inhibition of beta-site APP cleaving enzyme (BACE)1 that seems to protect endothelial cell integrity in the context of CADASIL and AD (Chacón-Quintero et al., 2021). In the same way, rodent models of aging suggest that the mitochondrial overexpression of catalase improves diminish vascular impairment and benefits neurovascular coupling (Csiszar et al., 2019).

On the other hand, uncontrolled post-stroke neuroinflammation, especially excessive microglial activation, is associated with cognitive deficits (Guruswamy and ElAli, 2017; Gefen et al., 2019; Jayaraj et al., 2019; Zhang et al., 2021). In this regard, strategies aiming to mitigate neuroinflammation *via* modulation of microglia could attenuate cognitive decline related to stroke (Guruswamy and ElAli, 2017; Dzyubenko et al., 2018; Jayaraj et al., 2019). In this regard, evidence suggests that the prominent microglial regulator, insulin-like growth factor (IGF)-1, reduces gliosis while preserving brain volume and myelination, as well as motor performance and memory when administered intranasally in aged mice (Farias Quipildor et al., 2019). Efficient immunomodulatory approaches capable of fine-tuning microglial activation are still to be developed.

Importantly, the emergence and turnover of stroke-mediated proteinopathies are associated with BBB disruption and neuroinflammation. Although the role of cSVD is critically important in the etiology of VaD, there is still a huge gap in the literature as to our understanding of the underlying mechanisms. This is due to the fact that the current knowledge is obtained either from genetic or non-clinically relevant sporadic animal models. Indeed, the majority of cSVD cases are sporadic and associated with diverse risk factors, as such, it is critically important to develop animal models that replicate some of the pathological features associated with VaD. However, the overwhelming findings indicate the loss-of-function on the local cerebrovascular network caused a central role in initiating a pathological cascade of events that lead to altered neuro-glial functions, and thus subsequently dementia (Rouhl et al., 2012; Shoamanesh et al., 2015; Fu and Yan, 2018; Li et al., 2018; Jian et al., 2020).

Air pollution has emerged as a significant risk factor for cerebrovascular and neurodegenerative disorders (Wu et al., 2015; Åström et al., 2021). It is becoming clear that exposure to PM, one of the most deleterious air pollutants, increases the risk of chronic neuroinflammation that leads to dementia (Wu et al., 2015; Åström et al., 2021). Cell-based assays have demonstrated that PM acts as a powerful inflammatory and oxidative stress mediator in various brain cell types. Interestingly, PM exposure seems to amplify the pathological responses underlying VaD pathobiology. Although the impact of PM on neurovascular functions is evidenced *in vitro*, little is known about its role in mediating neurovascular impairments *in vivo*. Future studies should consider investigating the consequences of PM exposure as a comorbid condition in the design of preclinical experiments.

As previously mentioned, besides PM, various modifiable vascular risk factors are recognized to impact VaD. For instance, stroke and cSVD share common risk factors such as hypertension, atherosclerosis, obesity, atrial fibrillation, diabetes, dyslipidemia, high homocysteine, metabolic syndrome, smoking, as well as cardiac and carotid arterial disease (Barnes and Yaffe, 2011; O’Brien and Thomas, 2015; Kalaria et al., 2016; Tariq and Barber, 2018). Given their central role, various preventive approaches have been developed and adopted to attenuate the impact of this triad of vascular on VaD. Such approaches are exemplified by the landmark multidomain Finnish Geriatric Intervention Study to Prevent Cognitive Impairment and Disability (FINGER), which has been shown to enhance all cognitive sub-domains through a multidomain lifestyle intervention that include dietary counseling, physical exercise, cognitive training, and vascular and metabolic risk monitoring for a period of 2 years (Ngandu et al., 2015; Kivipelto et al., 2020). Furthermore, knowing the detrimental role of high blood pressure in VaD prevalence, the Systolic Blood Pressure Intervention Trial (SPRINT) and its sub-study the Memory and Cognition in Decreased Hypertension (MIND) were established with an emphasis on investigating the consequences of lowering systolic blood pressure. Although the incidence of dementia was not improved, the trial reported a reduction of mild cognitive impairment (MCI) and MCI composite in subjects with lower blood pressure. Interestingly, these observations were associated with a reduction of WM lesion volumes (Kjeldsen et al., 2018; Peters et al., 2019). Despite that, each trial discloses important limitations, yet the impact of modifying main VaD risk factors was shown to allow the development of efficient interventions. Further research is needed for longer periods with a more representative population to lessen the limitations and improve the efficiency of interventions aiming to attenuate dementia prevalence.

Finally, a deep analysis of the current scientific knowledge outlines brain pericytes as a major effector in the pathobiology of VaD. Through their spatial localization, pericytes play a central role in integrating and processing signals from their milieu to generate critical neurovascular functions, which include BBB maintenance, CBF modulation, vascular stabilization, and immunomodulation (Zlokovic, 2011; Hermann and ElAli, 2012). Following a stroke, pericytes undergo cell death at the ischemic core and get activated in the peri-lesion site where they detach from the vasculature (Zlokovic, 2011; Hermann and ElAli, 2012). Importantly, it has been reported that even after successful recanalization, pericytes located at the peri-lesion site remain contracted impeding the capillary microcirculation, which leads to vascular constriction and chronic hypoperfusion (Dalkara, 2019). Moreover, microcirculation of the white matter was disrupted upon pericyte degeneration leading to the accumulation of toxic blood-derived fibrotic deposits within the vasculature, which promotes vascular fibrosis, accompanied by a reduction in the regional CBF (Montagne et al., 2018). These changes are supposed to be directly implicated in the pathogenesis of diffuse WMD associated with loss of oligodendrocytes and subsequently myelinated axons (Montagne et al., 2018). The findings indicate that pericyte degeneration plays a major role in the pathogenesis, and thereby therapies, of WMD associated with cSVD (Montagne et al., 2018). Furthermore, the generation of pericytes is translated by elevated levels of the soluble-platelet derived growth factor receptor (PDGFR)β (sPDGFRβ) in the CSF, strongly correlating with BBB breakdown, CBF reduction, and cognitive decline (Sweeney et al., 2020). Therefore, decoding the pericyte reactivity to stressors related to the vascular risk factors constitutes a promising avenue that might lead to achieving major breakthroughs in getting new mechanistic insights in the pathobiology of VaD and in developing novel therapeutic interventions.

## Author Contributions

The authors contributed to the review as follows: SL wrote, edited, and finalized the draft as well as prepared the figures. DM-C wrote, edited, and finalized the draft as well as prepared the figures. YE wrote the section related to air pollution. AEA conceptualized, wrote, and finalized the draft. All authors contributed to the article and approved the submitted version.

## Conflict of Interest

The authors declare that the research was conducted in the absence of any commercial or financial relationships that could be construed as a potential conflict of interest.

## Publisher’s Note

All claims expressed in this article are solely those of the authors and do not necessarily represent those of their affiliated organizations, or those of the publisher, the editors and the reviewers. Any product that may be evaluated in this article, or claim that may be made by its manufacturer, is not guaranteed or endorsed by the publisher.
